# Improving medical students' attitudes towards the chronic sick: a role for social science research

**DOI:** 10.1186/1472-6920-10-84

**Published:** 2010-11-22

**Authors:** Kenneth Mullen, Malcolm Nicolson, Philip Cotton

**Affiliations:** 1 Academic Unit for Mental Health and Wellbeing, School of Medicine, University of Glasgow, 1st Floor Admin Building, Gartnavel Royal Hospital, 1055 Great Western Road, Glasgow G12 0XH, UK; 2 Centre for the History of Medicine, Lilybank House, Bute Gardens, University of Glasgow, G12 8RT, UK; 3 Section of General Practice and Primary Care, School of Medicine, University of Glasgow, UK

## Abstract

**Background:**

Many medical students are negatively disposed toward the elderly and chronic sick. The present study assessed the impact of a community-based teaching initiative, the Life History Project, on students' attitudes to these groups.

**Methods:**

A questionnaire including Likert based responses and free text comments was distributed to all first-year MBChB students after completion of their Life History coursework. Data was analysed using SPSS and content analysis.

**Results:**

A high proportion of students believed the Life History Project had increased their understanding of both psychological and social aspects of health and illness and the role of the humanistic social sciences within this. We discovered that the Life History Project not only gave students first-hand experience of the elderly and chronic sick but also had a positive effect on their attitudes towards these groups. The qualitative free text comments corroborated these views.

**Conclusions:**

It is possible to positively influence medical students' attitudes towards these stigmatised groups; it is therefore important that we continue to enhance opportunities for learning about the impact of chronic illness on individuals and society throughout the curriculum.

## Background

The purpose of medical education is to produce good doctors. The attributes of the good doctor have recently been helpfully explored by Macnaughton [1]. She emphasises the centrality of a training in biomedicine - both to provide the medical student with an understanding of how the body works and to inculcate an appreciation of the nature and importance of research. But the complete, humane practitioner requires more that biological science. As Macnaughton puts it, 'Doctors need ... to be able to assimilate the scientific knowledge of disease and treatments with the understanding of the individual patient.' To achieve this understanding requires a degree of insight into the contexts of patients' lives. Macnaughton argues that students can benefit, in this respect, from the study of art, philosophy and literature. That is undoubtedly true. However, given that the views and attitudes of individuals are largely shaped by their social experience, and that much ill-health is strongly determined by social environments, one might conjecture that the humanistic social sciences would also have a valuable role to play in medical education. This paper reports the results of incorporating an element of social science teaching and research training within the first year of the medical curriculum at Glasgow University.

The aim of the 'Life History Project', as it is called, is to help first-year medical students develop an understanding of the impact of long-term illness on people's lives. This is achieved by means of a short exercise in qualitative research, in which the students conduct semi-structured interviews with chronically ill patients in their own homes. Patients are allocated to students by general practitioners who work as tutors in Glasgow University Medical School. The GP tutors select, brief, and obtain consent from, suitable subjects from their patient lists. The patients are not deliberately chosen according to criteria of socio-economic deprivation but, given the prevalence of poverty in the city of Glasgow and the economic handicaps often suffered by those with chronic illness, many of the selectees exhibit economic and social disadvantage. The majority are elderly.

For reasons of safety and mutual support, students are allocated in pairs to each interviewee. Two interviews are conducted, the first with the patient with a chronic illness in his or her own home, and the second with a significant other person, ideally a principal carer. The interviews carried out by students in their visits are guided by qualitative semi-structured interview schedules similar in style to those used in standard qualitative research. The students also take field notes about their interviewee's socio-economic circumstances and environment.

Before going into the community to undertake the interviews, the students are given a taught introduction to the social perspective on health and medicine. This is followed by a brief review of the methods of qualitative social research and, in particular, the techniques of semi-structured interviewing. A 'role play' practice in semi-structured interviewing is undertaken in which students interview one another about some aspect of their own experience of illness and healthcare.

After the community-based interviews have been completed, a 'de-briefing' session is held in the School of Medicine, facilitated by two humanistic social scientists (KM and MN), in which the students first discuss their findings in small groups and then present the common themes that they have identified to the class as a whole. They are briefed on the importance of confidentiality and anonymity, and provided with suggestions for background reading in social science as it applies to medicine. Each student then submits a written report, in which they analyse the content of the two interviews, and give an account of their observations and impressions of the patient and his or her circumstances. The reports are summatively assessed.

The aim of our research was to discover if student experience of the Life History Project had made them more positively disposed towards the chronic sick and elderly, and towards the behavioural sciences in medicine.

## Methods

In 2008, it was decided to gauge the impact of this exercise on students' attitudes. Ethical approval having been obtained, a questionnaire was devised to elicit student opinion of the Life History Project (LHP). Our study questionnaire was developed from course evaluation instruments used within the medical and dental schools to evaluate the MBChB and the BDS teaching courses. The questionnaire was first piloted with a sample group. A few minor alterations were made to its wording, and that of the accompanying information sheet, to improve clarity. Then, six weeks after submission of the coursework (by which time the summative results had been released), the questionnaire was distributed to all first-year students. The questionnaires were distributed and collected by KM and an administrative assistant. Students were asked to read the information sheet and, if they were willing to take part in the study, to sign an acceptance sheet. The questionnaires were completed anonymously so as to minimise the danger of students seeking to give socially desirable answers. 227 questionnaires, from a class of 240, were returned.

Participants entered their responses to the various Likert items on EDPAC multiple choice sheets which were later scanned by an optical mark reader, while they gave their answers to open-ended questions as free-text comments in a space provided on the questionnaire sheet. The Likert attitudinal items were pre-coded and analysed using SPSS 15.0. The free-text comments were analysed using a content analysis approach [2]. This was enhanced by using the framework method and constant comparison [3,4]. Identified themes were compared across the data, and interpretations discussed within the team.

In the section below we will first present the qualitative findings and then bring the quantitative results into the discussion.

## Results

### Qualitative Analysis

Students were asked three open-ended questions: 1) 'What did you like best about the Life History project?' 2) 'What did you like least about the Life History project?' and 3) 'What improvements to the Life History project could you suggest?' Following on from this, analysis identified five themes as follows.

Students appreciated the genuineness and realism of the experience. They were able to practice their communication skills in a 'natural environment' away from the institutional medical context and the safe settings of actors and simulated patients.

'Going to interview the patient which helped develop communication skills in a "real" environment.' (1)

'Meeting the patients face to face without the supervision of our tutor, which allowed us to improve/practice our communication skills in a natural environment.' (2)

They believed that they had gained insight into the person's life and how illness impacts upon it.

'I liked being given the opportunity to gain insight into the life of a person suffering from a chronic illness.' (3)

'Being able to see a chronic illness in context with the whole person i.e. not just learning about it by textbook, but to be able to see its impact and consequences in real life.' (4)

The Life History Project had made them begin to think about the psychological and social effects of disease. They could appreciate the different dimensions of chronic illness, environmental and social factors, and how an illness, or illnesses, impacted upon their interviewee's everyday life. Students also appreciated the degree of independence the Life History Project gave them.

'Visiting a patient's home and focusing on the less medical factors influencing their health such as social interaction. It allowed me to appreciate various factors that contribute to a patient's health particularly relating to physical and built environments.' (5)

'The part I liked best was the interviewing of the patient in their home environment which helped me to understand the ways in which they live and how it impacts on their chronic illness.' (6)

'The chance to speak to a real life patient, rather than a simulated one, and discuss the nature of their illness/disability and how their social circumstances allow or do not allow them to come to terms with and cope with their illness/disability.' (7)

Moreover, they appreciated having their stereotypes challenged and seeing the patient as a person.

'The most positive aspect of the LHP for me was being able to see and understand more about the life of someone with a chronic illness "from the other side of the looking glass", in seeing how it affected their personal life, beyond the medical aspects seen from the point of view of a doctor.' (8)

'It was a good experience to understand the impacts of chronic illness by interviewing a patient rather than reading about the possible effects in a textbook.' (9)

The Life History Project stimulated the students to think of the psychological and social effects of disease. It enhanced their appreciation of the different dimensions of chronic illness, environmental and social factors, and how illness fitted in with normal life. They began to see common themes among patients with chronic illness: loss of independence and the burden on carers; multiple morbidity; the importance of social networks; the effect of differing psychological attitudes, positive or negative, and uncertainty in relation to the course of an illness.

Some students felt an awkwardness when asking some questions which were seen to be too personal, and they became emotionally concerned at a person's deteriorating lifestyle.

'We visited, we revisited and we thanked the person, it felt a little rude, we should revisit over a longer period of time.' (10)

Some students suggested that there could be an increased number of interviews either by talking to more patients who were chronically ill, or to a wider range of people:

'A chance to speak to more people involved in the patient's care e.g. neighbours.' (11)

'I think that more visits could be useful' (12)

'I think in the future the use of two different patients would be useful and a comparison could be made, although this may take too much time.' (13)

Writing up the project proved a challenge for some. For students this could mean a wish for a greater word limit to be set to the final report:

'Bearing in mind that many of the patients are elderly and suffering from a chronic disease, they will most likely have extensive medical histories. A bigger report should be allowed to be written in order to convey everything in as clear and accurate details as does the patient justice.' (14)

'Having such a tight word limit; as I thought it was hard to portray somebody's life and situation in less detail than I would have wanted, or thought needed, to create the image of the patient as a whole.' (15)

Some students wished to see more clinical detail and access to people's medical history and notes before conducting the interviews.

'More information about the patient given beforehand so students could formulate individual questions.' (16)

### Quantitative analysis

The thirteen variable data-set was obtained from answers to specific attitudinal items using a five point Likert scale, ranging from 'strongly agree' to 'strongly disagree'. As in standard research practice [5-7], these scales were then dichotomised for clarity. The numbers strongly and weakly agreeing, as against being neutral, weakly disagreeing, or strongly disagreeing, are presented in Table 1.

**Table 1:** Medical students' attitudes towards dimensions of the Life History Project

	Strongly agree and agree	Neutral, disagree and disagree strongly	Total
I am now aware of how the social aspects of a person's life affect how they deal with illness.	91.2% (207)	8.8% (20)	100% (227)

I am now aware of the role patient's attitudes (either positive or negative) have in mediating the impact of an illness on their lives.	89.4% (203)	10.6% (24)	100% (227)

The project made me more aware of the dynamics of chronic illness.	86.3% (196)	13.7% (31)	100% (227)

The project has increased my knowledge of the interaction between the psychological and the social in relation to health and illness.	85.4% (193)	14.6% (33)	100% (226)

I feel the project has helped me with my communication skills.	75.3% (171)	24.7% (56)	100% (227)

The life-history project has increased my understanding of the medical humanities.	74.8% (169)	25.2% (57)	100% (226)

My empathy towards client groups such as the elderly and the chronic sick has improved.	62.4% (141)	37.5% (85)	100% (226)

The project has helped with my ability to take a medical history.	63.0% (143)	37.0% (84)	100% (227)

The project has increased my understanding of research methods.	21.6% (49)	78.4% (178)	100% (227)

Overall I found the project enjoyable.	70.9% (161)	29.1% (66)	100% (227)

It is clear, from these results, that the Life History Project is succeeding in its object of encouraging students to think more deeply about the social and psychological aspects of ill health. We would wish to highlight the top two attitudinal items of the table as being particularly salient for our conclusions. 91.2% of participants said they had become more aware of how the social circumstances of a person's life affect how chronic illness is coped with, whereas 89.4% agreed that that they had become more aware of the role that individuals' attitudes have in mediating the impact of illness. These conclusions are strongly supported by the qualitative data. In Quote 8 above, for example, a student speaks appreciatively of the deeper understanding of an individual patient that participation in the Project has brought. Quotes 5, 6 and 7 all indicate that knowledge of the social and domestic circumstances of particular patients has lead to an improved general understanding. 62.4% of participants believed that their empathy with the elderly and the chronic sick, in general, had improved.

It should be noted that these attitudinal changes occurred in the context of an exercise that the majority of students (70.9%) found enjoyable and worthwhile. As already noted, students valued the genuineness and realism of the experience, taking place away from the safe setting of actors and simulated patients, and free from the constraints of the medical school environment. The project also had other perceived benefits. 75.3% felt the project had helped enhance their communication skills, and 63% believed the project had improved their ability to take a medical history.

The free-text responses to Questions 2 and 3 on the questionnaire, which sought to elicit negative and constructive criticism respectively, also indicate positive attitudes to the Life History Project. As Quotes 10 to 16 above indicate, students asked for more prolonged contact with, and deeper knowledge of, the patients and their carers. Some students evidently became emotionally involved with their interviewee and his or her restricted or deteriorating lifestyle. On occasion, however, awkwardness was felt when asking personal questions. One disappointing finding was that only 21.6% of participants felt that the involvement in the LHP had improved their understanding of research methods.

The evidence of the questionnaire is also supported by the content of the students' final written reports, in which they typically display sensitivity to a number of key aspects of the psycho-social model of illness. These routinely include the importance of family relationships and social networks, of economic and housing issues; and of standards of both formal and informal care. Students have also emphasised the significance of stigma, the association between social isolation and depression, and the emotional burden imposed upon familial carers. The prevalence of multiple morbidity among the elderly has been regularly remarked upon.

## Discussion

Morrison has highlighted the fact that many medical students are negatively disposed toward treating the elderly and the chronically sick [8,9]. This is a matter of serious concern. Not only should the members of these client groups receive the same standard of care as the rest of the community, their numbers are set to increase considerably in the coming decade or two, posing great challenges to the National Health Service [10,11]. However our experience with the Life History Project in Glasgow would seem to suggest that, under the right circumstances, students can develop more positive attitudes, and that experience with a method of social science research, namely semi-structured interviewing, can aid that process.

This may seem a surprising conclusion given that medical students can often be unreceptive to input from the behavioural and social sciences [12]. A recent survey by Litva and Peter concluded that, in the United Kingdom, 'the implementation of B&SS [behavioural and social science] teaching in medical education remains highly problematic' [13]. However, one of the keys to the success of the LHP would seem to be its location, not just in the community but in the patient's own homes. The immediacy of the contact with patients that this provides seems to be highly valued by first-year students.

The importance of community-based learning for undergraduate medical students is increasingly recognised. Dirnan et al note that early experience in the community setting 'makes learning more real and relevant' [14]. Pill and Tapper-Jones describe a project that successfully gives students contact with the community at the very beginning of their undergraduate careers [15]. Direct interaction with patients has also been found to improve attitudes towards stigmatising diseases [16]. However many of these initiatives merely substitute one formal institutional setting, a medical school classroom or a hospital ward, for another, a general practitioner's surgery or a health care facility. We would argue that learning about the impact of chronic disease, about its personal, economic and social implications, is more vivid if done in the patient's own environment, where it is possible to acquire a deeper knowledge of an individual's circumstances and lifestyle. Given that most medical students are from relatively comfortable and affluent socio-economic backgrounds [17], and very many of the patients that they will encounter in their professional lives will be less privileged, it is important that, right from the start of their careers, that they are appreciative of the importance of learning to communicate across such social divides. The greater immediacy of personal contact within an informal domestic setting, we believe, encourages and engenders empathic understanding.

We would also argue that the LHP provides evidence to endorse two of suggestions made by Litva and Peters as to how best to enhance the possibilities of successful social science education within a medical curriculum. One is that students are more likely to develop a positive attitude to social science education if it is fully integrated with learning about the practice of medicine. Glasgow's LHP is facilitated by staff in the Section of General Practice and Primary Care, and students are briefed about their interviewees by general practitioners who work as tutors for the Section. The link this makes with medical practice in the community would seem to be important in enhancing the students' opinion of the LHP. Litva and Peters also point to the crucial importance of committed, high-quality teaching. Both KM and MN are trained humanistic social scientists, who are experienced, specialist educators in this field.

Another difference between the LHP and other community-based educational initiatives is the emphasis that we have placed on the students regarding themselves as social science researchers engaged in a primary research project. Many of the other initiatives that have involved the ill laity in medical education have chosen to encourage the students to view the patients they meet as 'teachers' [18-20]. Undoubtedly much success has been achieved with this approach. However we believe that a cultivation of the researcher role enhances the status of the exercise in the student's mind, provides valuable training in research methods (even if this is not fully appreciated by the students), and is also more in keeping with the ethos of a problem-based learning curriculum. We would also contend that the apparent failure of the students to gain an enhanced appreciation of the research process was belied by the quality of the report work that the students finally submitted.

The students undertaking of the Life History Project are at a very early stage of their undergraduate medical careers, just four months into their studies. But such experience is reinforced in their second and third years with similar and connected projects utilising elements of qualitative approaches: the family project and the community diagnosis project [21]. There is every reason to believe therefore that this introduction to elements of qualitative methodology has beneficial effects, and is further reinforced as they undertake additional, complementary projects in years two and three of their studies.

We are not aware that patient's attitudes to their involvement in the LHP are adversely affected by our emphasis on research, or by the domiciliary context of our interviews, although that might be a suitable subject for further investigation. It is heartening, in this respect, that assessments of patients' responses to involvement in medical education have indicated that it is, overwhelmingly, a positive experience [20].

We also believe that the educational experience is enhanced by the students interviewing a principal carer as well as the chronically ill patient. Wee, Davies and Holt have recently described a programme, run from the Sobell Study Centre in Oxford, in which students in their penultimate year of medical school, participate in a workshop with bereaved lay carers [22]. The authors note that 'carers carry a wealth of experience and observations which could provide powerful and authentic learning'. Moreover, 'rarely is the caregiver's welfare considered in its own right'. Interviewing the carer, in the patient's home, often with the patient present, can thus be an intense learning experience. As noted previously, participating students are sensitised to the emotional burdens of constant care. However some have also recognised the fulfilment that caring can provide under favourable circumstances. In future we may wish to expand the educational value of the Life History Project by having the students interview other members of the patient's social network.

We are of course aware of the limitations of our data; particularly in relation to not having information on the students' views prior to the introduction of the Life History Project, which would have acted as a direct comparison. However the work of other authors [8,9,12,13] has established that medical students generally hold negative views not only about the chronic sick and elderly but also in relation to the importance of the medical humanities.

## Conclusion

The present study therefore shows that it is possible positively to influence medical students' attitudes to the elderly and the chronic ill-health. However, as Jha and his colleagues have recently cautioned, changes of attitudes early in a course are not necessarily carried over into its final years, far less into professional practice [23]. The 'erosion of empathy' as the student progresses through medical school is a well-documented phenomenon [24,25]. However, in Glasgow University, the Life History Project is complimented by a number of other community-based curriculum components throughout the five years which, it is hoped, sustain and reinforce an awareness of the importance of understanding each patient, whether chronically or acutely ill, as an individual, in his or her own social context [21]. We would argue that the results presented in this paper substantiate the case that social science education has a valuable role to play in this crucial educational process.

## Competing interests

The authors declare that they have no competing interests.

## Authors' contributions

KM designed the questionnaire, carried out the first preliminary analysis of the data, and produced the first draft of the paper. PC and MN were then involved in revising the manuscript, with MN, at a later stage, undertaking a significant second redraft. All authors read and approved the final manuscript.

## Pre-publication history

The pre-publication history for this paper can be accessed here:

http://www.biomedcentral.com/1472-6920/10/84/prepub
